# Histological observation of a gelatin sponge transplant loaded with bone marrow-derived mesenchymal stem cells combined with platelet-rich plasma in repairing an annulus defect

**DOI:** 10.1371/journal.pone.0171500

**Published:** 2017-02-08

**Authors:** Xiang Xu, Jianzhong Hu, Hongbin Lu

**Affiliations:** 1 Department of Spine Surgery, Xiangya Hospital, Central South University, Changsha, PR China; 2 Key Laboratory of Organ Injury, Aging and Regenerative Medicine of Hunan Province, Changsha, PR China; 3 Department of Sports Medicine, Research Centre of Sports Medicine, Xiangya 10 Hospital, Central South University, Changsha, PR China; Mayo Clinic Minnesota, UNITED STATES

## Abstract

**Objective:**

To research the histological characteristics of a gelatin sponge transplant loaded with goat BMSCs (bone marrow-derived mesenchymal stem cells) combined with PRP (platelet-rich plasma) in repairing an annulus defect.

**Method:**

BMSCs were separated from the iliac crest of goats, sub-cultured and identified after the third generation. Then, PRP was obtained using blood from the jugular vein of goats via two degrees of centrifugation. In the animal experiments, the goats were divided into the following three groups: a sham group, an injury group and a therapeutic group. In the sham group, we decompressed the lamina and exposed the annulus fibrosus. In the injury group, we exposed the annulus fibrosus after decompression of the lamina and created a 1 × 1 cm defect in the annulus using surgical instruments. In the therapeutic group, after decompression of the lamina, we exposed the annulus, created a 1 × 1 cm defect using surgical instruments, and placed a gelatin sponge combined with BMSCs and PRP into the defect for a combined method of repair. Three, six and twelve weeks after the surgery, the previously damaged or undamaged annulus tissue was removed from the three groups. Then, the above tissue was assayed using HE (hematoxylin-eosin) staining, Masson trichrome staining, AB-PAS (Alcian blue-periodic acid Schiff) staining, and type II collagen staining and observed by microscopy.

**Results:**

From the HE staining, we observed that the number of repair cells gradually increased. Compared to the injury group, the cell density and gross morphology of cells in the therapeutic group were closer to those of the sham group. As observed by Masson trichrome gelatin staining, many of the fibroblast cells or tissues were under repair, and as time progressed, the number of fibroblast cells and amount of tissue gradually increased. The results of the AB-PAS staining suggest that chondrocytes participated in the repair of the annulus. The level of type II collagen gradually increased, as determined by immunohistochemical staining.

**Conclusion:**

Our results demonstrate that a gelatin sponge transplant loaded with BMSCs and PRP can effectively repair annulus defects.

## Introduction

In recent years, the number of patients with lumbar disc herniation has increased each year. Lumbar disc herniation has become a common disease, affecting the quality of daily life [1]. Minimally invasive surgery to remove the nerve nucleus and treat lumbar disc herniation can be used to reduce the suffering of these patients [2]. However, once the nerve nucleus is removed, the surrounding annulus tissue around the nucleus will also be destroyed. As a result, lumbar disc herniation may be recurrent, and the nucleus will project from the damaged annulus and press on the nerve [3,4]. To prevent recurrence, the easiest solution is to suture the annulus injury. Using goats, Ahlgren [5] showed that suturing only the nucleus pulposus could provide a certain amount of support, preventing the nucleus from protruding; however, this method cannot be used to compensate for the tissue that has been lost or for the sutured part of the fibrous ring. Biocompatibility has been very poor; however, tissue engineering has received much attention in recent years. Tissue engineering in treatment often uses a scaffold, which can achieve good biocompatibility and good degradation. Furthermore, a scaffold can help repair fiber ring damage and can simulate the structure of the fiber ring under normal conditions.

Therefore, the annulus fibrosus may be repaired using tissue engineering methods to reduce the recurrence rate of surgery. Bone marrow-derived mesenchymal stem cells (BMSCs) have become indispensable seed cells in tissue engineering because of their ease of extraction, rapid proliferation, and multiple differentiation potential, among other characteristics [6,7,8,9]. Moreover, third-generation BMSCs grow very well [10]. Platelet-rich plasma (PRP) [11,12,13] has been used in therapeutic treatment to control inflammation and to enhance the repair of musculoskeletal injuries. The use of PRP has recently grown in popularity. PRP also acts as a stimulating factor to generate cytokines and growth factors, which are needed during cell proliferation to stimulate the proliferation of BMSCs. Furthermore, gelatin sponges were chosen as a tissue engineering scaffold in order to implant cells into annulus defects because they are sterile, water-insoluble, malleable, and absorbable. Gelatin sponges are easily obtained and inexpensive and are not known to induce allergic reactions or other harmful side effects; moreover, they have several other characteristics such as good histocompatibility, high porosity, osteoporosity and other organizational structures that help cells to grow and proliferate.

In this study, the intervertebral disc annulus fibrosus of a goat animal model was damaged, and the annulus defect was repaired using goat BMSCs combined with PRP, loaded into a gelatin sponge. After 3, 6, and 12 weeks, the repaired tissue was removed and the morphological characteristics were observed by hematoxylin and eosin (HE) staining, Masson trichrome gelatin staining, AB-PAS staining, and the detection of type II collagen in order to determine whether this method can repair the damaged annulus and to clarify which cells are involved in the repair process.

## Materials and methods

All experimental procedures conformed to the Chinese National Health and were approved by the Ethics Committee of the Center for Scientific Research with Animals and the Inner Mongolia Medical College biomedical research ethics review (Permit Number: 2012004). A total of 6 chinese male goats (6 months of age, 23–25 kg) were enrolled in the study (Laboratory Animal Center of Mongolia Agricultural University). The animals were housed individually under specific pathogen-free conditions in identical environments (temperature 22–24°C; humidity 60–80%) and were fed standard rodent chow ad libitum with unlimited food and water. All of the goats were anesthetized using an intramuscular injection of xylazine hydrochloride at a concentration of 1 mL/5 kg (Kermel, Tianjing, China). After surgery, the goats were housed individually in cages at a constant temperature with free access to food and water and were monitored twice a day. A pain relief drug (Tramadol, Grunenthal GmbH, Aachen, Germany) and an antibiotic (penicillin sodium, North China Pharmaceutical Co., Shijiazhuang, China) were administered once a day for the first three days after surgery. The bladders were manually expressed twice daily until full voluntary or autonomic voiding was obtained. All efforts were made to minimize suffering. All of the animals were healthy, and there were no unintended deaths of animals throughout this study. BMSCs were provided by the Laboratory of Molecular Pathology, Inner Mongolia Medical College.

### Isolation, culture and identification of goat BMSCs

**Fig 1:**
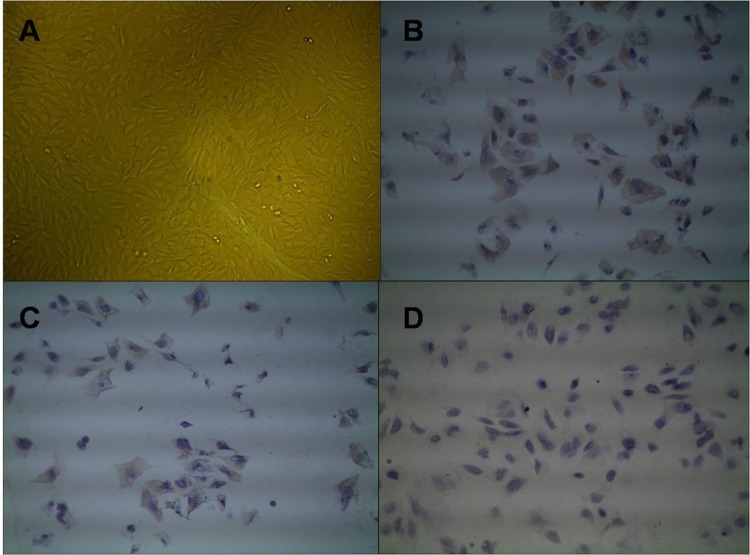
The morphological characteristics and identification of third-generation BMSCs. (A) Third-generation BMSCs (×100). (B) Third-generation BMSCs that were CD44-positive (×100). (C) Third-generation BMSCs that were CD29-positive (×100). (D) Third-generation BMSCs that were CD44-negative (×100).

After an intramuscular injection of xylazine hydrochloride II, the 6-month-old goats were placed on an operating table to ensure that they were in a lateral position. The bilateral iliac crests of the goats were punctured, and then, a bone needle connected to a syringe with 1 mL of heparin was used to extract 4 mL of bone marrow fluid (plus 1 mL heparin, total 5 mL). The BMSCs were isolated and purified using density gradient centrifugation and an adherent culture. We then adjusted the cell concentration and seeded the cells in flasks for culturing. The BMSCs were observed, and photos were taken using an inverted microscope each day. The culture medium was changed every other day. When the cells had reached a confluency of more than 85%, they were sub-cultured. When the goat BMSCs grew to the third generation, they were seeded in 6-well plates. The expression of CD29, CD34 and CD44 surface antigens on the cells was detected using an immunohistochemical method, and the cells were identified (Fig 1).

### Preparation of PRP

**Fig 2:**
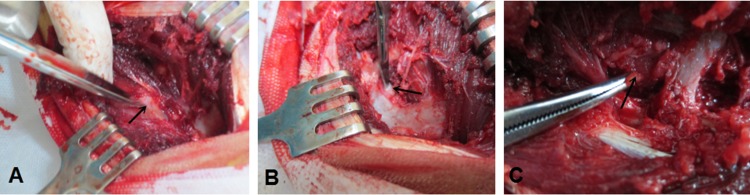
The exposure of annulus fibrosus after decompression of the lamina, (A) sham group, arrow showing annulus fibrosus. (B)injury group,arrow showing a 1 × 1 cm defect of annulus fibrosus. (C)therapeutic group,arrow showing complexes including BMSCs, PRP,and Gelatin sponges.

A 6-month-old goat was locally anesthetized at the jugular vein with lidocaine. Under sterile conditions, we opened PRP preparation packages from Weigao, connected a 50-mL syringe containing 5 mL of sodium citrate with lancets, and extracted 45 mL of goat blood. We placed all of the liquid from the syringe (50 mL, including the 45 mL of goat blood and 5 mL of blood sodium citrate) into a tube and centrifuged it with Weigao's special centrifuge (4000 r/min, 10 min). After centrifugation, we removed the red blood cells in the lowest layer at the interface to approximately 3–5 mm. The rest of the solution was centrifuged again (4000 r/min, 10 min) after trimming. After centrifugation, approximately ¾ of the supernatant was drawn and discarded. The remaining solution within the centrifuge tube is PRP. To better exert the effects of PRP, we mixed 1 mL of CaCl2, 10 million U of thrombin, and the above PRP to prepare a PRP jelly (Fig 2), using special a package from Weigao for subsequent assays.

### Experiment design

Six goats were enrolled and divided into three groups: a sham group (group A), an injury group (group B) and a therapeutic group (group C). Thirty discs from each group were used (n = 30), for a total of 90 discs. In the sham group (group A), the intervertebral discs of the goats were not subjected to any treatment. In the injury group (group B), the goats were placed on an operating table in a lateral position after an intramuscular injection of xylazine hydrochloride II. The intervertebral discs, thoracic 1 to lumbar 5, were targeted for surgery. We shaved a surgical field with a 15-cm radius using shaving shears and protected the region from infection by applying povidone-iodine 2–3 times. Sterile towels were placed on the hole, and the four corners were fixed with towel forceps in order to expose the surgical field. The longitudinal skin located 2–3 cm from the spinous process of the goat was opened using scissors to make a 10- to 15-cm incision, and the skin was opened layer by layer with a four-claw retractor lift to expand the operating field. The hemostasis method and a hemostat were used to stop bleeding during the surgery. During the surgery, two ribs were located, in the middle of which was a white nodule called the transverse process. The white 1-cm coil located below the front end of the transverse process is the annulus. The periosteum and soft tissue were peeled using periosteal stripping to create a 1 × 1 cm gap breakage on the annulus with a knife. The other discs in the injury group were also subjected to the same treatment. After the surgery, the goats were given Soviet Union 3 with twice the dose of anesthetic to cause them to awake sooner. Half an hour later, the goats woke quickly and stood up. The goats were injected with 10 million u penicillin daily for 5 days to prevent infection. The therapeutic group (group C) received the same treatment as group B. After damaging the annulus with a 1 × 1 cm gap using a knife, we combined third-generation BMSCs and PBS in a cell suspension with a gelatin sponge (1 × 1 cm) in a sterile environment. The gelatin sponge was implanted into the hole, combined with PRP-like jelly above the gap (Fig 2). At 3, 6 and 12 weeks after the surgery, the annulus tissue that had been previously damaged or undamaged was removed from the three groups, the histological tissue was evaluated, and the annulus tissue growth in each group was observed by microscopy.

#### HE staining and criterion for evaluation

The animals were deprived of feed, but were allowed free access to water for 24 h before being sacrificed. At the end of sacrificing, blood was collected, and the annulus fibrosus was removed from the goats and fixed in 10% formalin. After fixation, the tissues were processed and embedded in paraffin. The paraffin blocks were cut into 4-μm sections and stained with Harris’ hematoxylin and eosin for histological observation under a microscope.

The paraffin sections were scored according to a modified pathological Mankin score with a graded assessment, which had a maximum score of 14. The following criteria were used for scoring: normal: 0–1; mild: 2–5; moderate: 6–9; and severe: 10–14. According to the above standard, the specimens were divided into a sham group, mild degeneration group, moderate degeneration group and severe degeneration group. After scoring, the data were analyzed.

#### Masson trichrome gelatin staining

The sections were stained as described by Sheehan and Barbara (1980). Intervertebral disc tissues were stained in Wiegert’s iron hematoxylin solution (50 mL of alcoholic hematoxylin and 50 mL of acidified ferric chloride solution) for 10 min. The tissues were then rinsed in tap water for 10 min and stained with Southgate’s mucicarmine solution (1 g of carmine, 1 g of aluminum hydroxide, and 100 mL of 50% ethanol) for 30 min to stain goblet cells, followed by rinsing in deionized water, staining in metanil yellow solution for 30 to 60 sec, and rinsing in distilled water.

The paraffin sections were scored according to a reference standard for the density of collagen fibers, based on the percentage of collagen fibers throughout the total area. The data were recorded and analyzed after scoring.

#### AB-PAS staining

Intervertebral disc tissues were stained with Alcian blue and periodic acid-Schiff reagent (Luna, 1968) using an Alcian blue solution (1 g of Alcian blue, pH 2.5, 3 mL/L of acetic acid, and 97 mL of distilled water) for 30 min to stain goblet cells. The staining was followed by rinsing in tap water for 10 min, oxidizing in periodic acid (5 g/L) for 5 min, rinsing in lukewarm tap water for 10 min, and staining in Coleman’s Schiff reagent as a counter stain (Sigma Chemical Co., St. Louis, MO) for 10 min.

#### Immunohistochemistry of type II collagen and quantitative determination of collagen

A sample of 0.5 mL of nucleus pulposus was diluted with acetic acid to 50 mL. Then, 20 mL of the mixture was centrifuged at 4000 r/min for 10 min for immunohistochemistry. Type II collagen expression was detected using a goat anti-mouse type II collagen antibody (Cell Signaling Technology, USA) and a type II collagen immunohistochemistry kit (Jingmei Biological Engineering Co., Ltd., China) and was observed under a microscope.

Paraffin sections were grouped and observed with a high-power microscope. Five fields were observed from each slice; we counted 100 cells per field. If the section was not colored, it was counted as 0 points; pale yellow was counted as 1 point; yellow was counted as 2 points; and tan was counted as 3 points. Using the percentage of positive stained cells, when positive cells occupied < 5% of all accounted cells, 0 points were recorded. A percentage of 5% to 25% positive cells was counted as 1 point; 26% to 50% positive cells were counted as 2 points; 50% to 75% positive cells were counted as 3 points; and more than 75% positive cells were counted as 4 points. The final criteria were produced from the above two scores, and the data were analyzed.

#### Statistical analysis

All data were statistically analyzed with SPSS 13.0 software. The data are shown as the mean ± standard deviation. Analysis of variance was used to compare the data between groups. Values were considered significant if P < 0.05.

## Results

### Morphology and identification of goat BMSCs

Primary goat BMSCs were generally adherent within 24 h and reached more than 85% confluency after 5–7 days. Throughout sub-culture, the BMSCs proliferated rapidly, the growth direction of most of the cells was irregular, and the morphology was angular or spindle-like under an inverted microscope. Nevertheless, the third-generation goat BMSCs grew in the same direction as the colony growth and fusiform morphology (Fig 1). Cell surface antigens of the third-generation goat BMSCs were detected, and it was found that the expression of CD29 and CD44 was positive, with most of the cytoplasm being brown and the nucleus being purple. In the identification of CD34, the expression of CD34 was negative, most of the cytoplasm was not brown, but rather violet in color, and the nuclei were purple (Fig 1). These results suggest that the extracted cells were purified goat BMSCs, rather than other bone marrow cells.

### Histological observation and analysis of HE staining

**Fig 3:**
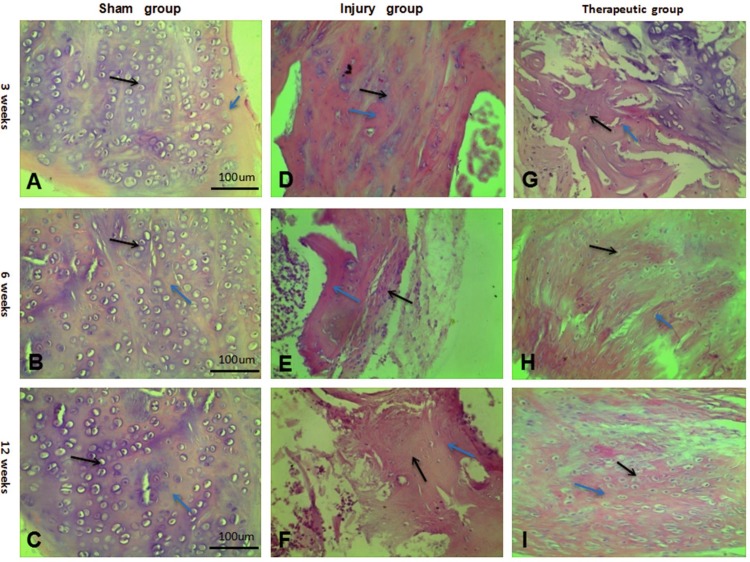
Results of HE staining in each group after 3,6,12 weeks. (A-C) HE staining in the sharm group after 3,6,12 weeks (×100). (D-F) HE staining in the injury group after 3,6,12 weeks (×100). (G-I) HE staining in the therapeutic groupafter 3,6,12 weeks (×100).Blue arrows:collagen and matrix.Black arrows:cartilage cells.

In the sham group, the bone cells and cartilage cells were dense with complete and normal morphology without any treatment; the nuclei were stained blue-purple, and cells were clearly observed after 12 weeks under light microscopy (Fig 3). In the injury group, where the gap was not repaired, a small amount of bone and cartilage cells appeared after 12 weeks. The cells were small with large nuclei and were surrounded by a small amount of collagen and matrix. There was a small number of fibroblasts with red dye, and proliferation of the fibrous tissue was not obvious. The general morphology included unclear cell figures and tissue structures. In the therapeutic group, due to our combined repair method, many bone cells and cartilage cells had proliferated with a large, clear vesicular morphology and were stained to exhibit a blue-violet nucleus. The arrangement and growth of the cells were normal, and the cells were surrounded with a small amount of collagen and matrix after 12 weeks. The gap had been filled with collagen and matrix, which had been secreted by cells, and the integrity of the tissue structure was nearly identical to that of the sham group. Meanwhile, a large number of fibroblasts and fibrous tissue had proliferated to repair and fill in the defective regions (Fig 3).

According to the Mankin pathological scoring standard, a low score was maintained for the sham group at 3, 6, and 12 weeks. Because the sham group had not received any treatment, the value tended to be normal (Table 1). Our data show that in the injury group, without any repairs, the defects underwent repair, but the value was significantly different from that of the normal tissue after 12 weeks (P < 0.05), belonging to the moderate degeneration group. However, the score for the therapeutic group decreased in a stepwise manner and nearly reached that of the sham group after 12 weeks, with statistical significance (P < 0.05). This group was labeled as a mild degeneration group, suggesting that the annulus had been successfully repaired and that our defect treatment had been effective (Table 1).

**Table 1:** HE staining score for each group (x ± s, n = 90).

Group	Time		
	3 weeks	6 weeks	12 weeks
**Sham group**	2.000 ± 1.054	2.000 ± 0.820	2.200 ± 1.033
**Injury group**	11.300 ± 1.494	9.800 ± 1.620	8.700 ± 1.703
**Therapeutic group**	9.100 ± 1.370	6.200 ± 1.400	3.400 ± 1.350
***P* value**	< 0.05	< 0.05	< 0.05

### Histological observation and analysis of Masson trichrome gelatin staining

**Fig 4:**
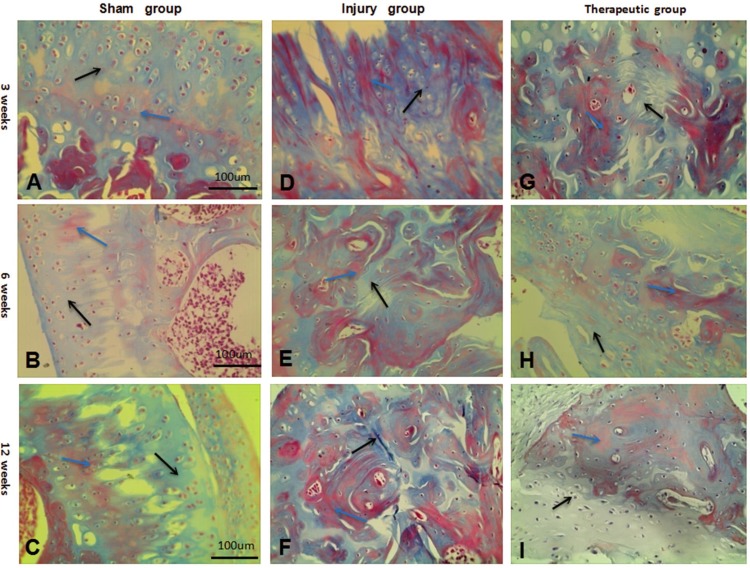
Masson staining in each group after 3,6,12 weeks. (A-C) Masson staining in the sharm group after 3,6,12 weeks (×100). (D-F) Masson staining in the injury group after 3,6,12 weeks (×100). (G-I) Masson staining in the therapeutic group after 3,6,12 weeks (×100). Blue arrows:mature bone trabecular.Black arrows:muscle fiber connective tissue.

In the sham group, which had no intervention, a large number of cartilage cells stained with a red nucleus and blue cytoplasm were observed after 12 weeks under light microscopy. Collagen fibers and the cartilage matrix were stained blue. The lacunae were not colored and were surrounded by blue-stained mature bone trabecular and red-stained immature bone trabecular, which exhibited a generally normal morphology. In the injury group, the defects remained unrepaired after 12 weeks, a small amount of cartilage cells was arranged in a disorderly manner, and cells were small with deep stained nuclei and a small amount of pale blue collagen fibers and cartilage matrix. Bone lacuna was surrounded by a large number of immature red-stained bone trabecular, around which was a small amount of muscle fiber stained red. The cell morphology was incomplete, and the repair status was not ideal. In the therapeutic group, after 12 weeks of joint repair, a large number of vacuolated chondrocytes appeared with large cells exhibiting blue-stained nuclei. The cells grew regularly and densely, surrounded by cartilage matrix and collagen fibers stained blue. The bone lacuna was surrounded by a large amount of mature blue-stained trabecular and a small amount of immature red-stained trabecular. Mature trabecular was surrounded by a large amount of red-stained muscle fiber connective tissue to repair the defects. The histological structure of the therapeutic group tended toward the normal structure of the sham group, with a complete morphology, suggesting that our defect repair was effective (Fig 4).

For the score of collagen fiber density, the analysis results showed that a high score was maintained in the injury group after 3, 6, and 12 weeks, corresponding to the score value for normal tissues without any treatment. For the injury group, without any repair for the gap, the collagen fiber density tended to increase over time, but the increase was slow and the reparation was not sufficient. The results for the injury group were significantly different from those for the sham group (P < 0.05). For the therapeutic group, the collagen fiber density tended to increase rapidly with time and exhibited a significant difference compared to the injury group (P < 0.05), suggesting that the repair function was more effective. After 12 weeks, the density value was significantly different from that of the injury group (P < 0.05) (Table 2), showing that the tissue was successfully repaired.

**Table 2:** Masson staining score for each group (x ± s, n = 90).

**Group**	Time		
	3 weeks	6 weeks	12 weeks
**Sham group**	64.700 ± 2.869	65.300 ± 2.869	64.400 ± 3.2041
**Injury group**	33.800 ± 2.530	36.100 ± 3.348	38.600 ± 1.506
**Therapeutic group**	36.800 ± 2.820	53.200 ± 3.553	56.200 ± 2.658
***P* value**	< 0.05	< 0.05	< 0.05

### Histological observation of AB-PAS staining

**Fig 5:**
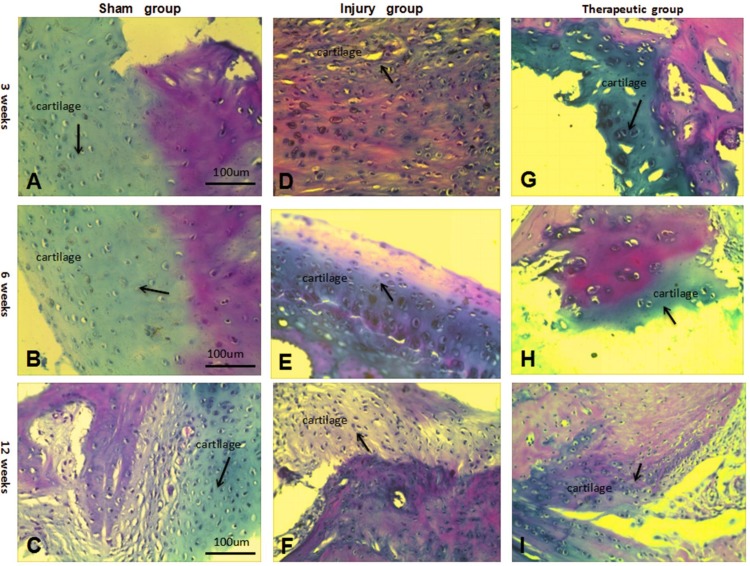
AB-PAS staining in each group after 3,6,12 weeks. (A-C) AB-PAS staining in the sharm group after 3,6,12 weeks (×100). (D-F) AB-PAS staining in the injury group after 3,6,12 weeks (×100). (G-I) AB-PAS staining in the therapeutic groupafter 3,6,12 weeks (×100). Black arrows:cartilage cells.

In the sham group, a large number of cartilage cells of normal tissues were observed after 12 weeks under light microscopy; these cells were arranged in line and were columnar in shape. The cartilage matrix appeared irregular and was stained blue, with clear boundaries between the cartilage and the bone plate. In the injury group, after 12 weeks, a small number of cartilage cells were aggregated and arranged in a disorderly manner, and there were no clear boundaries between the cartilage and the bone plate. A small amount of red-stained sticky sugars secreted by the cartilage matrix was present. The morphology was still largely defective and not completely repaired. In the therapeutic group, after 12 weeks of combined repair, a large number of vacuolated chondrocytes appeared with a large volume; the chondrocytes were columnar, were tightly arranged and had blue-stained nuclei. The cells surrounding the cartilage matrix were stained deep blue and tended to be mature. The boundary between the cartilage and the bone plates was not clear, and the cartilage plates formed a severe concave impression toward the bone plates, indicating the repair process. The cartilage matrix was surrounded by a high level of sticky sugars stained deep red, which repaired the tissue (Fig 5). The tissue structure was generally similar to that of the sham group, with a normal structure and complete morphology, showing that tissue was successfully repaired.

### Detection and analysis of type II collagen

**Fig 6:**
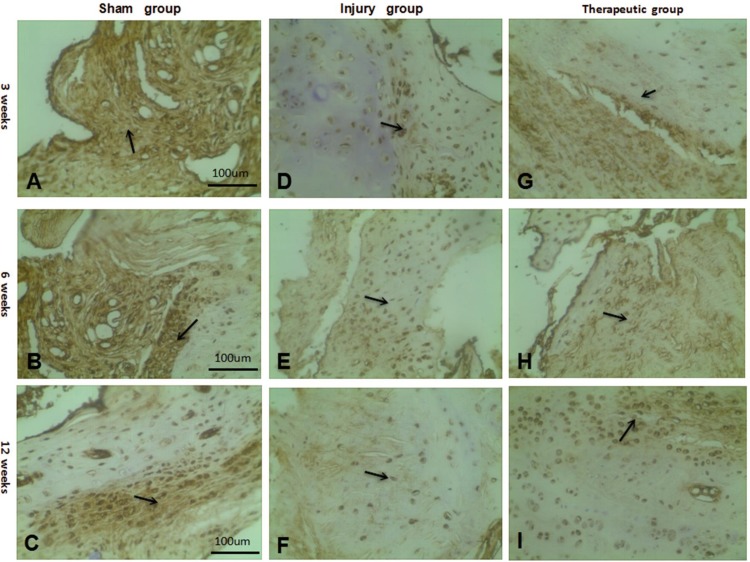
The results of type II collagen staining in each group after 3,6,12 weeks. (A-C) Type II collagen staining in the sharm group after 3,6,12 weeks (×100). (D-F) Type II collagen staining in the injury group after 3,6,12 weeks (×100). (G-I) Type II collagen staining in the therapeutic groupafter 3,6,12 weeks (×100). Black arrows: type II collagen.

In the sham group, after 12 weeks, a large number of cells were observed under light microscopy to have deep yellow-stained cytoplasm and a secreted matrix. The type II collagen staining showed a strongly positive result. In the injury group after 12 weeks, a small amount of yellow-stained particles was observed due to the absence of treatment. Both the cytoplasm and the secreted extracellular matrix were stained light yellow, and the arrangement of the cells was loose and irregular. The type II collagen staining results showed a weakly positive result. In the therapeutic group after 12 weeks, a large number of cells were stained deep yellow, as observed under microscopy, due to the joint repair procedure. The cytoplasm and the secreted extracellular matrix were both stained yellow, and the cells were tightly arranged and grew in a defined direction. Type II collagen was stained with a positive result (Fig 6). The general structure was similar to the morphology of the sham group, with similarity in the staining pattern, indicating that the tissue was successfully repaired.

The slices were scored, and in the sham group, without any treatment, the type II collagen level was at a normal high level. In the injury group, as time progressed, the level of type II collagen increased, but the rate of increase was small. After 12 weeks, the results were significantly different from those of the sham group (P < 0.05). Thus, the injured annulus group had undergone some repair, but not to a great extent, suggesting that the annulus was not completely repaired. In the therapeutic group, due to the effect of combined treatment, the type II collagen level increased over time; the score was significantly greater than that of the injury group (P < 0.05), indicating a high rate of repair in the therapeutic group. After 12 weeks, the type II collagen content of the injury group exhibited a small, but significant, difference (P < 0.05). With the ability to be fully repaired, these tissues had become like normal tissues (Table 3, Fig 6).

**Table 3:** Quantitative analysis of type II collagen staining for each group (x ± s, n = 90).

Group	Time		
	3 weeks	6 weeks	12 weeks
**Sham group**	7.800 ± 2.150	7.700 ± 1.567	7.900 ± 2.183
**Injury group**	2.600 ± 1.075	3.100 ± 1.449	3.600 ± 1.578
**Therapeutic group**	3.100 ± 1.370	4.400 ± 1.776	6.000 ± 1.944
***P* value**	< 0.05	< 0.05	< 0.05

## Discussion

Due to economic development, lumbar disc herniation has become a common disease in the general population. Water loss from the discs is gradually aggravated with age, and disc degeneration gradually increases. Severe disc degeneration causes a series of back and leg pain symptoms as well as lumbar disc herniation. Currently, there are only two ways to treat this condition: conservative treatment using acupuncture and a traction bed or surgery. To effectively alleviate the suffering of patients, surgery is an effective and feasible treatment. However, during the procedure, the annulus must be destroyed in order to remove the nerve nucleus. If the defective annulus is not repaired after surgery, the possibility of postoperative recurrence increases greatly, which can lead to psychological and physical suffering for the patient [4,14,15]. Therefore, a method for repairing the annulus has recently become a topic of great interest. Meanwhile, cell transplantation is a popular method in tissue engineering.

BMSCs are currently the most important seed cells in tissue engineering, and they have the potential to differentiate into multiple types of cells. Due to their convenience in extraction, rapid proliferation, and lack of antigen exclusion, etc., BMSCs are often used to repair bone, cartilage, muscle and other tissues. Numerous experiments have shown that BMSCs have the ability to differentiate into chondrocytes [16,17]; therefore, BMSCs were chosen to repair the defects in the tissue annulus in this study. Gradient centrifugation and adherent culture methods were used to isolate and purify the BMSCs and to identify the purified BMSCs while immunochemical methods were used to detect BMSC surface antigens.

To cause the BMSCs to exert their potential effects, PRP was selected as the stimulating factor of BMSCs in this study. PRP effectively stimulated the proliferation and differentiation of BMSCs, helped prevent a rejection immune response, and provided the ability for annulus repair. Furthermore, PRP can also secrete a variety of stimulating factors [18,19] that have the ability not only to stimulate the proliferation of BMSCs, but also to stimulate the secretion of mucopolysaccharides and collagen, which both function in tissue injury repair. In order to stably fix the seed cells in the annulus gap, the gelatin sponge was indispensable as a tissue engineering scaffold [20,21]. Gelatin sponges have several advantages such as good histocompatibility, porosity, osteoporosity and other organizational structures that help BMSCs grow and proliferate.

In this experiment, the discs were divided into the following three groups: a sham group, an injury group and a therapeutic group. Each group was treated independently, and the discs were removed after 3, 6 and 12 weeks to examine whether the tissues had been repaired. HE staining showed that the therapeutic group had an increase in the number of extended cartilage cells; moreover, more muscle fiber cells were observed over time, and the cells had become large with blue-stained vesicular nuclei [22]. In addition, the cartilage matrix, muscle fibers, collagen and other tissues that had differentiated were mature, and the gross morphological organization was similar to that of the injury group. It was demonstrated that the BMSCs had differentiated into a cartilage matrix, muscle fiber tissue, and collagen tissue in order to repair the annulus tissue [23]. Compared with the injury group, in terms of the number of cells, state, tissue differentiation and maturation degree, the extent of repair in the therapeutic group was far better, indicating that the injury group had not yet reached the final extent of repair.

Masson tricolor gelatin in the therapeutic group after 3, 6, and 12 weeks showed an extension of cartilage cells, and the myofibroblasts significantly increased the cartilage cell volume, vacuolization, and orderly growth. The cartilage matrix and collagen tissue area exhibited an increase in blue staining, as well as an increase in the area with red-stained muscle fibers and mature trabecular and an increased degree of repair. Compared with the injury group, the therapeutic group displayed a higher extent of repair. Moreover, the general trend of the injury group was based on the ability to repair muscle fibers and strengthen collagen fibers. Based on the data, the surface collagen fiber density in the group increased over time. It is suggested that the differentiation of BMSCs into tissue was not only in form, but also in content, as the tissue successfully repaired the annulus tissue defects [24].

The results of AB-PAS staining showed that in the therapeutic group, there was an increase in the chondrocyte packet over time as well as increases in the number and volume of cells, the amount of Aizen in the nucleus, and the extent of mature cell differentiation. AB-PAS staining was visible in a general form in the cartilage, and the bone plate was ill-defined; in females, the bone cartilage growth plate indicated that the differentiation of BMSCs into cartilage helped repair the defect in the annulus. The surrounding cartilage contained a large amount of blue-stained cartilage matrix, which secreted an abundance of red-stained sticky sugars. Mucopolysaccharides are a necessary type II collagen repair tissue. Thus, the differentiation of BMSCs into a fibrous ring of cartilage matrix has the ability to help repair the defect. Compared to the injury group, based on the growth of cells and tissues and their morphology, the extent of repair in the therapeutic group was better and more desirable than in the injury group.

In regard to the detection of type II collagen, the therapeutic group stained deeper and more yellow than the injury group, the number of yellow particles was greater and the particles were more orderly. According to rating standards, a deeper level of dyeing and a higher level of type II collagen indicate normal levels. The level of type II collagen was higher in the therapeutic group compared to the control.

This study presents a bioremediation method for increasing the expression of type II collagen content to promote a stronger and deeper repair of annulus tissue. The level of type II collagen in the therapeutic group was closer to that of the injury group, which may explain how the differentiation of BMSCs into type II collagen aided in successful repair of the tissue annulus [25].

In conclusion, our results demonstrate that a gelatin sponge transplant loaded with BMSCs and PRP can effectively repair annulus defects. This study provides a reliable method for protection from the recurrence of surgery lumbar disc herniation, which may provide a potential therapeutic method in the future.
